# Pathogen load and species monitored by droplet digital PCR in patients with bloodstream infections: A prospective case series study

**DOI:** 10.1186/s12879-022-07751-2

**Published:** 2022-10-04

**Authors:** Ziqiang Shao, Jingwen Zhu, Yanyan Wei, Jun Jin, Yang Zheng, Jingquan Liu, Run Zhang, Renhua Sun, Bangchuan Hu

**Affiliations:** Emergency and Critical Care Center, Intensive Care Unit, Zhejiang Provincial People’s Hospital (Affiliated People’s Hospital, Hangzhou Medical College), Shangtang Road 158, 310014 Hangzhou, Zhejiang China

**Keywords:** Bloodstream infection, Droplet digital polymerase chain reaction, Pathogen DNA load, Case series

## Abstract

**Background and objectives:**

Bloodstream infection (BSI) is a life-threatening condition in critically ill patients, but pathogen quantification techniques during treatment are laborious. This study aimed to explore the impact of monitoring pathogen DNA load changes and polymicrobial infection in blood by droplet digital polymerase chain reaction (ddPCR) on the prognosis of patients with BSIs.

**Methods:**

This prospective case series study was conducted in the general intensive care unit of the Zhejiang Provincial People’s Hospital and included patients with BSIs from May 2020 to January 2021. Pathogens DNA load and presence of polymicrobial BSIs were dynamically monitored by ddPCR.

**Results:**

Sixteen patients with BSIs proven by blood culture were recruited (87.5% men; mean age, 69.3 ± 13.7 years). All pathogens identified by blood culture were Gram-negative bacteria, among which seven were multidrug-resistant strains. The 28-day mortality rate was 62.5%. Compared to the 28-day survivors, the non-survivors were older (*P* = 0.04), had higher pathogen DNA load on the second (day 3–4) and third (day 6–7) ddPCR assay (*P* < 0.01 in both cases). In addition, the changes of pathogen DNA load in the 28-day survivors had a downward trend in the first three ddPCR assay, whereas stable load or an upward trend was observed in the 28-day non-survivors. Moreover, the number of pathogen species in patients with BSIs in the 28-day survivors decreased during the period of effective antibiotic treatment.

**Conclusion:**

The changes of pathogen DNA load and species monitored in blood by ddPCR may be used to determine antibiotic efficacy and make a more accurate prognostic assessment in patients with BSIs.

**Supplementary information:**

The online version contains supplementary material available at 10.1186/s12879-022-07751-2.

## Introduction

Bloodstream infection (BSI) is diagnosed by the presence of microbiologically positive cultures of blood from a patient with signs of systemic infection and is either a primary infection or secondary to an existing infection at another body site [1, 2]. BSI is a life-threatening disease in critically ill patients and is always associated with adverse outcomes due to delay appropriate antimicrobial treatment and source control [3, 4]. The choice of antimicrobials for BSI treatment in critically ill patients is routinely empirical at the beginning and then adjusted according to the results of blood cultures and antibiotic susceptibility tests [5, 6]. A daily re-evaluation of the effectiveness of the antibiotic regimen based on the available information is an important strategy during BSI treatment, especially during the first 72 h of treatment [7]. The dynamic monitoring in the changes of pathogen load in response to the antibiotic regimen is an exciting and straightforward option for assessing the therapeutic effectiveness during BSI treatment.

Currently, blood culture is the gold standard for identifying the pathogenic microorganisms in BSI, as it is easy to perform and displays an excellent analytical performance [8]. However, the rhythm imposed by the growth time requirements of blood culture is incompatible with the monitoring speed requirements during BSI treatment [9]. The initiation of an empirical antimicrobial therapy significantly increases the possibility of false negatives in blood cultures and precludes determination of antibiotic sensitivity [10]. Moreover, because it is impossible to measure microbial load routinely using broth culture, the dynamic monitoring during the treatment of BSI lacks direct pathogen quantification [11]. Thus, there is an urgent need to develop a rapid and quantitative method for monitoring the changes of pathogen DNA load during the treatment of BSI.

In a recent consensus statement, Timsit et al. [9] suggested that molecular diagnostics can accurately detect pathogenic microorganisms of BSIs, particularly Gram-negative bacteria. The droplet digital polymerase chain reaction (ddPCR) as a novel molecular diagnostic technique was applied to diagnose infectious diseases, and could accurately detect and quantify nucleic acids without generating a calibration curve [12–18]. In our previous studies, it has been demonstrated that ddPCR was faster (4.2 ± 1.5 vs. 49.3 ± 6.8 h) and more sensitive than metagenomic next-generation sequencing (mNGS) in detecting target pathogens and had certain advantages over mNGS in identifying drug-resistance genes in patients with BSIs [18, 19]. Owing to its advantages in identifying polymicrobial BSIs and the possibilities for dynamic monitoring of changes of pathogenic microorganisms in blood, ddPCR can be used to evaluate antibiotic efficacy and survival prognosis.

Therefore, application of ddPCR might be more optimal than blood culture for monitoring changes in pathogens during the treatment of BSIs. In this study, we explored the impact of pathogen DNA load and species monitored by ddPCR in blood on the prognosis and antibiotic efficacy in patients with BSIs in an intensive care unit (ICU).

## Methods

### Study design and patients

This prospective case series included patients with BSIs admitted in the general ICU of the Zhejiang Provincial People’s Hospital from May 2020 to January 2021. The inclusion criteria were (1) > 18 years of age and (2) positive blood culture. The exclusion criteria were (1) pre-existing BSIs during hospitalization, (2) negative ddPCR test results, (3) the pathogens isolated from blood culture were not consistent with those detected by ddPCR assay, (4) any terminal-stage disease. Upon the suspicion of BSI, whole blood samples were simultaneously obtained for blood culture and ddPCR assay. Follow-up blood cultures were performed at least once. The second and third ddPCR assays were performed on day 3–4 and 6–7, respectively, mainly based on evaluating initial empiric antibiotic efficacy and guiding de-escalation of antimicrobial therapy. This study was approved by the Institutional Review Board and Ethics Committee of the Zhejiang Provincial People’s Hospital (No. 2019KY002). Written informed consent was obtained from all patients. All data were anonymized prior to analysis.

### Blood culture and antibiotic susceptibility test

A set of 2 blood culture specimens was drawn from each patient according to routine clinical practice [20], with aerobic and anaerobic culture for each specimen. The blood cultures were incubated at 37 °C in a BacT/ALERT® 3D System (bioMérieux, France). Once the blood culture bottle flagged positive, Gram staining was performed, followed by subculture on a Columbia blood agar plate at 37 °C with 5% CO_2_. After incubation for overnight, colonies from the blood agar were subjected to matrix-assisted laser desorption ionization time of flight mass spectrometry (MALDI-TOF MS; VITEK® MS system, bioMérieux, France) for identification. Then the blood culture broth was inoculated into a commercial automated VITEK2 COMPACT system (BioMérieux, France) following the manufacturer’s protocols. The results of the antibiotic susceptibility test were interpreted according to the Clinical and Laboratory Standards Institute guidelines [21].

### Plasma DNA extraction and ddPCR

Peripheral venous blood (5 mL) was collected from each patient into an ethylenediaminetetraacetate-containing tube. Plasma was immediately isolated after centrifugation at 1,600 × g, and 22 °C for 20 min. DNA was extracted from 2 mL of plasma using a Magnetic Serum/Plasma DNA Kit (TIANGEN Biotech, Beijing, China), and the Auto-Pure 20B Nucleic Acid Purification System (Hangzhou Allsheng Instruments Company, Hangzhou, China) following the manufacturer’s protocol [22]. DNA was eluted in 50 µL of elution buffer and used for ddPCR assay promptly on the same day.

The ddPCR assay was performed using a Pilot Gene Droplet Digital PCR System (Pilot Gene Technology Company, Hangzhou, China) to detect 16 bacteria, 4 fungi and 4 antimicrobial resistance (AMR) genes following the manufacturer’s protocol as previously described [18]. The pathogens and AMR genes included in the ddPCR assay were shown in **supplemental Table 1**. Briefly, for each testing panel, the ddPCR master mix had a final volume of 15 µL comprising 1 × ddPCR premix, 1 µM forward and reverse primers, 300 nM each probe, 5 µL of isolated plasma DNA. After PCR amplification, droplets were analyzed using an iScanner 5 chip scanner (Pilot Gene Technology Company, Hangzhou, China). Data analysis for the droplet counts and amplitudes was performed with 30 min of hands-on time using GenePMS software version v2.0.01.20011.

## Data collection

A specific case report was used for data collection. Data regarding demographic and clinical characteristics (clinical profile, blood measurements, isolated pathogens, ddPCR-reported pathogens and DNA load, use of antibiotics, Acute Physiological and Chronic Health Assessment II [APACHE II] score and Sequential Organ Failure Assessment [SOFA] score), and 28-day mortality rate were collected within the first 24 h of BSI onset. Antibiotic combination therapy contained two or more antibiotics. Polymicrobial infection was defined as a disease caused by a mixed infection with two or more microorganisms.

### Statistical analysis

Data analysis was performed using SPSS 19.0 (IBM Corp., Armonk, NY, USA). Normally distributed continuous variables were presented as the mean ± standard deviation (SD) and analyzed using the Student’s *t*-test. Non-normally distributed continuous variables were presented as the median (P_25_, P_75_) and analyzed using the Mann-Whitney U-test. Categorical variables were presented as n (%) and analyzed using Fisher’s exact test. Effects were considered statistically significant if two-tailed *P*-values were below 0.05.

## Results

A total of 102 patients with suspected BSIs were consecutively recruited from May 1, 2020 to January 31, 2021. Among them, 16 cases were concordantly positive by blood culture and ddPCR, 49 tested positive only by ddPCR and 6 tested positive only by blood culture, and the remaining 31 cases tested negative by both blood culture and ddPCR. Therefore,16 patients with blood culture proven BSIs met inclusion and exclusion criteria, and were available for the final analyses. Of these 16 patients (87.5% men), all of them were septic patients, 3 were immunosuppressed, and 1 was a heart transplant recipient. Mean values for age and APACHE II scores were 69.3 ± 13.7 years and 26.9 ± 4.3, respectively. Multidrug-resistant infection, polymicrobial infection, and combination antibiotic therapy accounted for 43.8%, 37.5%, and 87.5%, respectively. The most common pathogen isolated by blood culture was *P*. aeruginosa (n = 7). Follow-up blood cultures remained positive in 3 patients, of whom one case initially tested positive for *P. aeruginosa*, but subsequently positive for *K. pneumoniae* in the second blood culture.

The 28-day mortality rate was 62.5%, and the mean survival time was 6.0 (5.0, 12.0) days in the non-survivors. The 28-day survivors (n = 6) and non-survivors (n = 10) had similar prevalence of immunosuppressive disease, and multidrug-resistant pathogens (*P > 0.05*). However, the survivors, compared with non-survivors, were younger (*P* = 0.04), had a lower prevalence of receiving mechanical ventilation (*P* = 0.04), and showed lower pathogen DNA loads on the second (day 3–4) and third (day 6–7) ddPCR assay (*P* < 0.01 in both cases) (Table 1).

**Table 1:** Characteristics of the patients with BSIs

Characteristics	28-day survivors (n = 6)	28-day non-survivors (n = 10)	*P*-value
Age (years)	60.2 ± 10.0	74.7 ± 12.9	0.04
Men, n (%)	4 (66.7)	10 (100.0)	0.13
Mechanical ventilation, n (%)	3 (50.0)	10 (100.0)	0.04
Use of vasoactive drugs, n (%)	4 (66.7)	10 (100.0)	0.13
Immunosuppression, n (%)	2 (33.3)	1 (10.0)	0.52
Combination antibiotic therapy, n (%)	6 (100.0)	8 (80.0)	0.50
Multidrug-resistant pathogens, n (%)	2 (33.3)	6 (60.0)	0.61
Polymicrobial BSIs, n (%)	1 (16.7)	5 (50.0)	0.31
DNA load in the first ddPCR (copies/mL), median (IQR)	247.0 (37.8, 1375.0)	191.0 (47.5, 2428.5)	0.79
DNA load in the second ddPCR (copies/mL), median (IQR)	0.0 (0.0, 106.8)	986.0 (248.0 2423.0)	< 0.01
DNA load in the third ddPCR (copies/mL), median (IQR)	0.0	3000.0 (812.0, 8426.0)	< 0.01
Laboratory investigation on the first day			
White blood cells, median (IQR) ×10^9^/L	11.0 (2.9, 39.4)	12.1 (4.9, 15.3)	0.88
Platelets, median (IQR) ×10^9^/L	91.0 (44.0, 131.7)	63.0 (38.5, 164.5)	0.90
C-reactive protein (mg/L), median (IQR)	87.9 (64.6, 191.1)	130.0 (70.7, 241.1)	0.37
Procalcitonin (ng/mL), median (IQR)	2.1 (0.7, 96.6)	5.6 (1.4, 31.0)	0.88
Laboratory investigation on the 2nd − 4th day			
White blood cells, median (IQR) ×10^9^/L	10.7 (3.1, 15.1)	8.9 (6.2, 10.8)	0.28
Platelets, median (IQR) ×10^9^/L	83.5 (35.3, 120.8)	32.5 (24.8, 82.5)	0.57
C-reactive protein (mg/L), median (IQR)	64.0 (45.9, 150.1)	185.0 (138.9, 237.2)	0.49
Procalcitonin (ng/mL), median (IQR)	1.3 (0.2,19.1)	19.7 (3.4, 46.0)	0.66
Laboratory investigation on the 5th − 7th day			
White blood cells, median (IQR) ×10^9^/L	10.4 (6.0, 13.9)	12.4 (4.0, 12.7)	0.38
Platelets, median (IQR) ×10^9^/L	100.0 (30.3, 165.0)	59.0 (4.5, 79.5)	0.67
C-reactive protein (mg/L), median (IQR)	45.7 (22.5, 85.1)	217.3 (176.2, 247.4)	0.18
Procalcitonin (ng/mL), median (IQR)	3.26 (0.27,7.52)	12.8 (4.0, 48.1)	0.26
APACHE II score	26.5 ± 9.6	27.1 ± 4.6	0.89
SOFA score	11.3 ± 4.4	10.8 ± 2.1	0.79

IQR, interquartile range; APACHE II: Acute Physiology and Chronic Health Evaluation II; SOFA: Sequential Organ Failure Assessment; ddPCR, droplet digital PCR; Values are presented as the mean ± SD, median (IQR), or number of subjects (percentage of the column total). Pathogen DNA loads on the third ddPCR assay were detected in 6 survivors and 5 non-survivors, respectively. ^a^The DNA load of blood culture positive pathogens by ddPCR assay

**Fig. 1:**
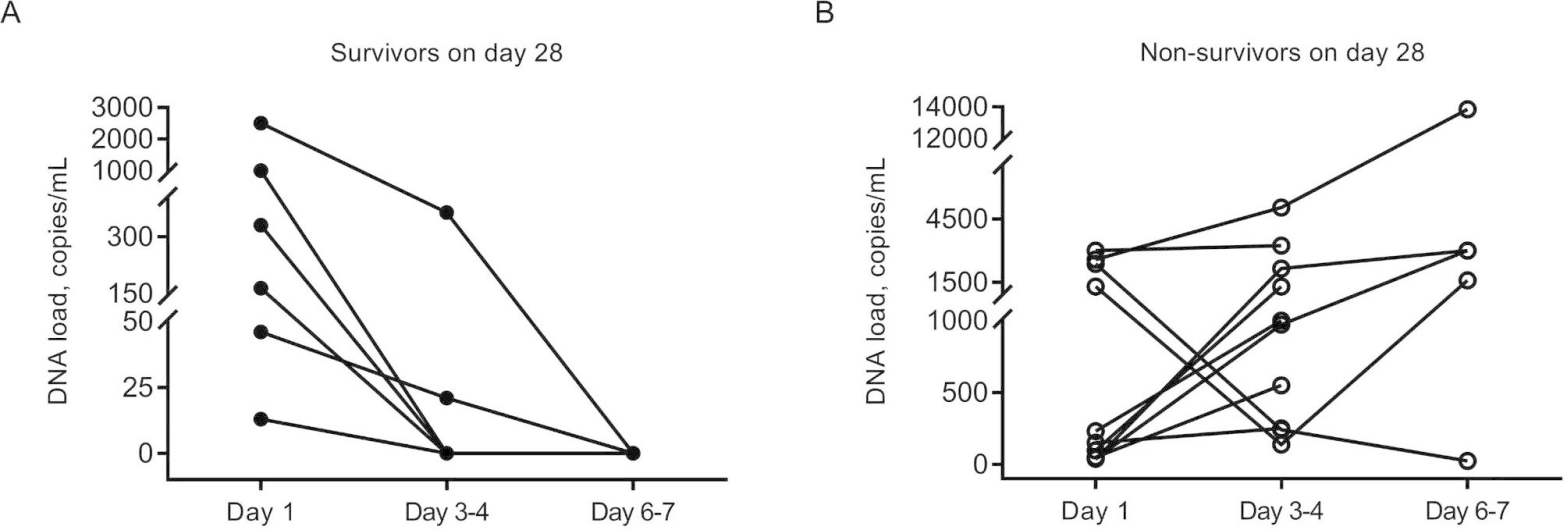
Changes in the DNA load of pathogens isolated by blood culture in the 28-day survivors (A) and non-survivors (B)

**Fig. 2:**
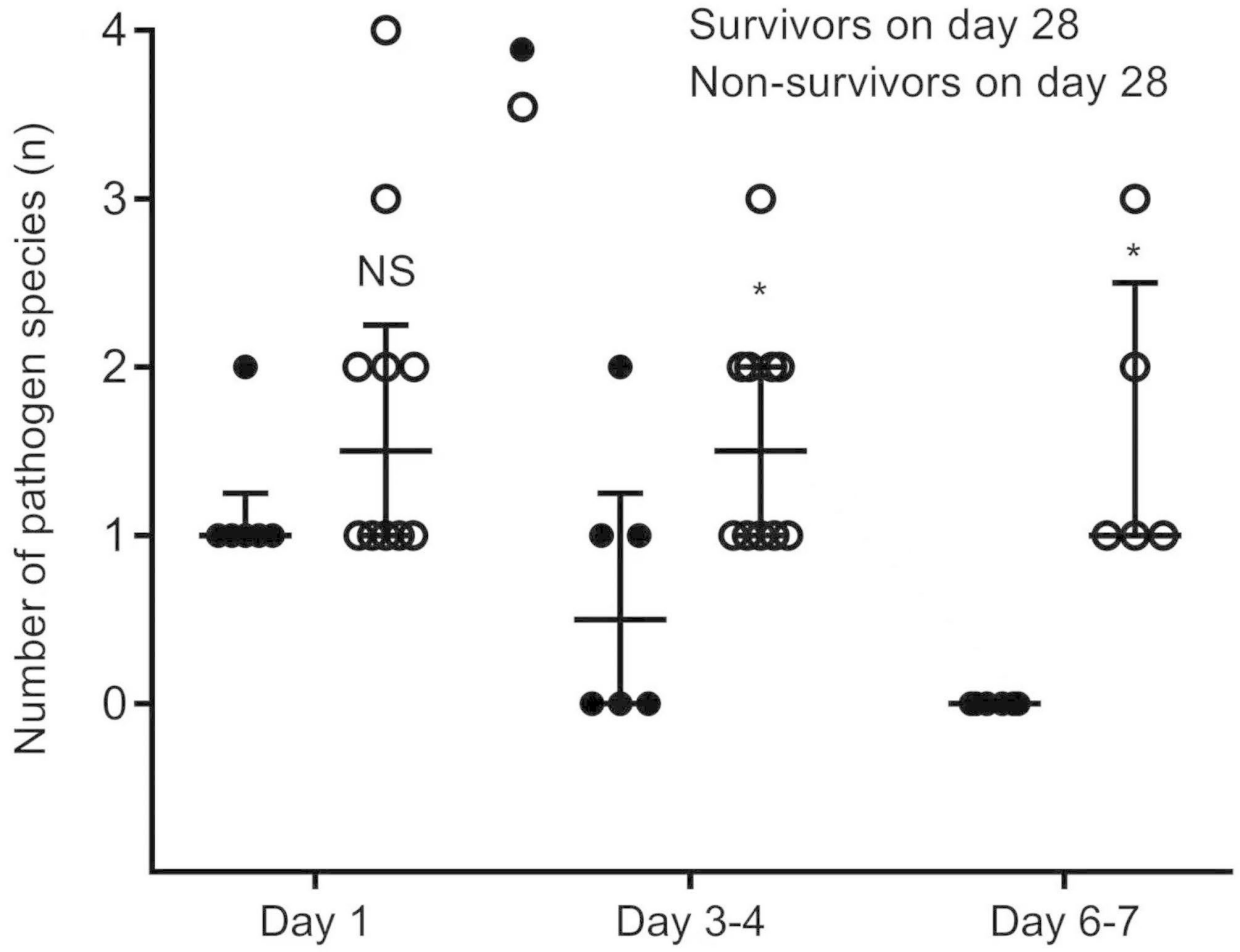
Differences in the number of pathogen species detected by ddPCR assay between 28-day survivors and non-survivors

Among the survivors, the pathogen DNA load detected by ddPCR showed a downward trend in all patients during treatment of antibiotics, and the pathogen DNA was detected negative on the third (day 6–7) ddPCR assay, whereas it showed an increasing trend in 9 of 10 non-survivors (Fig. 1). Among the non-survivors, one patient, who survived for 24 days, had an inconsistent trend between low pathogen DNA load trend and adverse outcome. In addition, six patients (37.5%) had polymicrobial BSI, of whom the survivors had a decreased number of pathogen species with effective antibiotic treatment. (Fig. 2).

Among the survivors, sensitive antibiotics were selected for the target pathogen identified by the first ddPCR assay. Non-sensitive antibiotics were stopped for 3 patients, and antibiotic regimen were unchanged for 2 patients. In addition, the four patients were performed for antimicrobial de-escalation therapy based on the negative result of the third ddPCR assay (Table 2). Among the non-survivors, 6 patients received inappropriate empirical antibiotic therapy after BSI onset, and the antibiotic regimen for 5 patients were not timely adjusted according to the results of the initial ddPCR assay (**Supplementary Table 2**).

**Table 2:** Adjustment of antibiotic regimen based on ddPCR assay in the 6 survivors

Case	Age (years) / sex		Comorbidities	Initial antibiotic regimen	Blood culture			First ddPCR assay (Copy numbers)	Adjustment of the antibiotic regimen based on first ddPCR assay		Third ddPCR assay (Copy numbers)	Days to de-escalation therapy	Antibiotic de-escalation regimen
1	65 / Male	Pulmonary embolism, Cirrhosis		Meropenem + Colistin + Caspofungin		*E. cloacae*	*E. cloacae* (1000)		Yes Meropenem + Colistin		Negative	14 days	Meropenem
5	53 / Male	Hypertension		Piperacillin/tazobactam + Linezolid		P. aeruginosa*	*P. aeruginosa* (64) *blaKPC* (641)		Yes Piperacillin/tazobactam + Colistin		Negative	7 days	Piperacillin/ tazobactam
7	71 / Male	Hypertension		Imipenem + Linezolid		*K. pneumonia*	*K. pneumonia* (2500)		No		Negative	7 days	Cefoperazone /sulbactam
10	49 / Female	Heart transplantation, ECMO, Immunosuppression		Meropenem + Colistin + Daptomycin + Caspofungin		*A. baumannii*	*A. baumannii* (164)		Yes Meropenem + Colistin + Caspofungin		Negative	12 days	Meropenem + Caspofungin
11	71 / Male	Hypertension, Post splenectomy		Piperacillin/tazobactam + Linezolid + Isepamicin		*P. aeruginosa*	*A. baumannii* (20) *P. aeruginosa* (13) *blaKPC* (67)			No	Negative	7 days	Piperacillin/ tazobactam
12	52 / Female	Acute lymphocytic leukemia, Immunosuppression		Meropenem + Caspofungin		K. pneumonia*	*K. pneumonia* (330) *blaKPC* (800)			Yes Ceftazidime/avibactam + Caspofungin	Negative	7 days	Discontinued

*blaKPC*, *Klebsiella pneumoniae* carbapenemase encoding gene; ECMO, Extracorporeal Membrane Oxygenation

* Multidrug-Resistant Organism, MDRO.

## Representative cases

Patient 1 was a 52-year-old woman hospitalized for acute lymphoblastic leukemia and received chimeric antigen receptor T-cell (CAR-T) immunotherapy in the department of hematology. She developed *Candida tropicalis* BSI during hospitalization. After 2 days of antibiotic treatment with Meropenem 1 g q8h combined with caspofungin 50 mg qd, the patient still developed septic shock and was transferred to ICU. Klebsiella pneumoniae carbapenemases (KPC)-producing *Klebsiella pneumoniae* was detected by ddPCR within 4 h on the first day of ICU admission, and subsequently the patient was treated with ceftazidime/avibactam 2.5 g q8h combined with caspofungin 50 mg qd. On the third day of ICU admission, the KPC-producing *K. pneumoniae* was isolated by blood culture and antibiotic susceptibility test. The only resistance gene encoding KPC, but not *K. pneumoniae* was detected by the second (day 4) ddPCR assay. On the seventh day, the third ddPCR result was negative, as was the second blood culture. Meanwhile, the patient’s hemodynamic status improved, and her body temperature recovered to be normal. All antibiotics were discontinued, and the patient was transferred out of ICU on the fourteenth day. Therefore, this case showed that ddPCR assay is more rapid (4 h vs. 2 days) and sensitive for target pathogen identification than blood culture, and can provide early initiation of accurate antibiotic therapy and guide antibiotic de-escalation therapy.

Patient 2 was a 62-year-old man hospitalized for chronic kidney disease, arthrolithiasis, and hypertension. Because of hypotension during dialysis, BSI was suspected, and the patient was transferred to the ICU. The patient was treated with imipenem 1 g q 6 h and linezolid 0.6 g q 12 h as initial antibiotic regimen. When admitted to ICU, blood culture and ddPCR assay were performed simultaneously. The first ddPCR assay reported that polymicrobial BSIs were present with *P. aeruginosa* (2000 copies per milliliter) and *K. pneumonia* (50 copies per milliliter), and the DNA load of *P. aeruginosa* was dominant. On the second day of ICU admission, blood culture only reported the presence of *P. aeruginosa*. According to the results of antibiotics susceptibility test, antibiotic regimen was adjusted to imipenem 1 g q 6 h and moxifloxacin 0.4 g q d. On day 4, blood culture and ddPCR assay were performed again. Although *P. aeruginosa* and *K. pneumonia* were both identified in the blood by ddPCR assay, the DNA load of these two pathogens has changed dramatically. The DNA copy number of *K. pneumonia* rose to 551 copies per milliliter, and became the dominant pathogen, whereas the DNA copy number of *P. aeruginosa* dropped to 97 copies per milliliter. On day 5, tigecycline 100 mg q 12 h was given to the patient based on the results of the second ddPCR assay. On day 7, the second blood culture concordantly reported the presence of KPC-producing *K. pneumoniae*. This case demonstrated that ddPCR can be used to dynamically monitor the change of pathogens DNA load to adjust antibiotic regimen in polymicrobial BSIs.

## Discussion

The main finding of the present study is that the dynamic monitoring of the changes in pathogen DNA load and the number of species in blood by ddPCR might help determining the efficacy of the initial antibiotic treatment in patients with BSIs and guide any necessary adjustments in therapy. Thus, ddPCR may be used to establish a more accurate diagnosis and improve prognosis of patients with BSIs.

In the present study, the DNA load of the pathogens was not significantly different between the 28-day survivors and non-survivors, when BSIs were diagnosed. It is similar to the observation in the study by Ziegler et al. [23] that reported an association of the initial pathogen DNA load with disease severity, but not mortality. Nevertheless, the results of several other studies on the molecular diagnosis of BSIs are inconsistent with our findings. In a prospective study of 27 adult patients with culture-proven *Staphylococcus aureus* bacteremia, Ziegler et al. [14] developed the ddPCR assay for 16 S rDNA and reported that non-survivors had significantly higher DNA load on days 1–2 than survivors (*P* = 0.03) [14]. Likewise, using the quantitative real-time PCR assay (qPCR), Ho et al. [24] observed that the levels of mecA DNA were significantly higher in the non-survivors than in the survivors at 0–2 days of MRSA bacteremia (5.48 vs. 4.58 log copies/mL, *P* = 0.003). Several reasons could account for the discrepancy between our observations and results of other studies. A fraction of the detected 16 S rDNA or mecA DNA can be derived from other bacteria, possibly due to polymicrobial or secondary infection. Indeed, the detection of 16 S rDNA and drug resistance genes indicate the presence of bacteria indirectly, lacking pathogen heterogeneity, whereas the ddPCR system directly targets nucleic acids of the pathogen. In addition, all these studies, including ours, had small sample sizes. Thus, further studies in larger samples are needed to determine the association between initial pathogen DNA load and mortality.

The changes in pathogenic DNA levels have been proposed as a potential surrogate prognostic marker in BSI assessments. In the present study, the pathogen DNA load decreased in all survivors and increased in nine of 10 non-survivors, which is in line with previous studies on the molecular diagnosis of BSIs [24, 25]. Indeed, in a prospective observational study that included 20 adult patients with culture-proven MRSA bacteremia, Ho et al. [24] reported that mean mecA DNA levels tended to decline continuously in the survivors. Likewise, in a prospective observational study of 51 critical patients with *A. baumannii* bacteremia monitored by qPCR, patients with a slower rate of initial bacterial clearance had higher in-hospital mortality than those with a higher clearance rate (Odds ratio: 2.32, *P* = 0.04) [25]. Moreover, the initial rate of bacterial clearance had good sensitivity and specificity in evaluating the appropriate antibiotic use. Taken together, our current results and data from previous studies suggest that the trend of the pathogen DNA load by ddPCR assay could potentially be used to monitor BSIs, which might be helpful in evaluating responses to therapy.

Polymicrobial BSIs have been reported for more than 50 years [26], with an incidence between 5% and 38% [27–29]. Compared with blood culture, ddPCR assay is more liable to detect polymicrobial BSIs, because it could eliminate the possible bias caused by the preferential amplification and enable quantification of low concentrations of pathogens. In the present study, polymicrobial BSIs were found in 37.5% (6/16) of the patients diagnosed by ddPCR, which was consistent with that reported in several previous studies [28, 29]. In addition, in this study, we observed that polymicrobial BSIs were associated with mortality. In line with this conclusion, a retrospective study that included 412 patients with bacteremia showed that patients with polymicrobial BSIs had higher 28-day mortality than those with monomicrobial BSIs (38.3% vs. 24.7%, *P* = 0.033). Moreover, the dichotomization of BSIs according to polymicrobial vs. monomicrobial appeared to have a prognostic value [30]. Likewise, in a retrospective case-control study, Pammi et al. [31] also observed that polymicrobial BSIs occurred in the neonatal ICU were associated with more than 3-fold increase in mortality (47% vs. 20%, OR = 4.3, *P* = 0.001) and an increase in duration of infection. Interestingly, in this study, we found a case of *P. aeruginosa* and *K. pneumonia* polymicrobial BSIs with a change in the dominant pathogen during the treatment of BSI monitored by ddPCR, which was validated by blood culture. The results of our study further demonstrated the potential value of ddPCR assay to guide antimicrobial therapy in monitoring polymicrobial BSIs.

Blood culture is the gold standard for detecting BSI pathogens, but it has some limitations. Due to relatively low sensitivity and a long turnaround time, it is difficult and impractical to optimize antibiotic regimen early based on blood culture results. Several recent studies have shown that molecular detection methods are more sensitive to monitor microbial change, and thus are helpful to timely optimize antibiotic regimens in patients with BSIs. In a prospective randomized controlled trial of 617 blood culture-positive patients, Banerjee et al. [32] observed that multiplex PCR assay combined with the real-time antimicrobial stewardship could shorten the time from Gram staining to appropriate antimicrobial escalation or de-escalation. In 14 patients with severe burns, mNGS testing has also been shown to have potential value in the diagnosis of polymicrobial BSIs and can provide accurate guidance for antibiotic therapy [33]. In this study, we observed that the changes in pathogens load and number of species determined by ddPCR have potential value for guiding the use of antibiotics in critically ill patients with BSIs. Additionally, our previous studies have demonstrated that ddPCR assay was more rapid (~ 4 h vs. ~2 days) and cheaper ($150 vs. $450) for identifying causal pathogens than mNGS testing [18, 19]. Therefore, ddPCR might be more suitable for the dynamic monitoring of pathogen changes during BSIs and implementation of real-time antimicrobial stewardship, although whether the ddPCR-guided antibiotic regimen improves the prognosis of patients with BSIs remains to be investigated.

This study should be interpreted with the context of limitations. First, the sample size was so small that the multivariable analysis could not be performed. Secondly, the ddPCR system only covered 20 common isolated pathogens and 4 antimicrobial resistance genes, and all the included cases had Gram-negative bacteria in blood culture, therefore the generalization of the results of our study should be cautiously interpreted. Thirdly, since some patients of non-survivors survived less than 3 days after BSI onset, follow-up ddPCR assay missed might affect the evaluation on efficacy of initial empiric antimicrobial therapy. In addition, for multiple pathogens detected by ddPCR, it is difficult to determine if a pathogen associated with a relatively low DNA load in blood is clinically relevant, especially if such pathogens were detected only once. Finally, this study was a single-center study, and the results should be validated using a multi-center study with a larger sample size.

In conclusion, the dynamic monitoring of pathogen DNA load and polymicrobial infection changes in blood by ddPCR helps to evaluate antibiotic efficacy and improves the accuracy of diagnosis of patients with BSIs. Therefore, ddPCR assay may be utilized to guide the adjustment of initial antibiotics and provide better prognosis of patients with BSIs. These conclusions, however, need further confirmation by larger studies.

## Electronic supplementary material

Below is the link to the electronic supplementary material.

Supplementary Material 1

## Data Availability

The datasets used and/or analyzed during the current study are available from the corresponding author on reasonable request.

## Declarations

### Authors’ information

Ziqiang Shao, Email: szqdoctor@163.com. Jingwen Zhu, Email: 914556550@qq.com. Yanyan Wei, Email: weiyanyan96@163.com. Jun Jin, Email: 13777816993@163.com. Yang Zheng, Email: zhengyang173@163.com. Jingquan Liu, Email: wsljq05@163.com. Run Zhang, Email: zrcindy@qq.com. Renhua Sun, Email: jqin168@hotmail.com. Bangchuan Hu, Email: hubangchuanicu@163.com.
