# Supplementary material for: Pathogen load and species monitored by droplet digital PCR in patients with bloodstream infections: A prospective case series study
Source: BMC Infect Dis. 2022 Oct 4;22:771. doi: 10.1186/s12879-022-07751-2 (PMC9531393; doi:10.1186/s12879-022-07751-2)
Supplement: Supplementary file 1 — Supplementary Material 1 [file 12879_2022_7751_MOESM1_ESM.docx]

**Supplementary Table 1.** Pathogens and AMR genes detection panels for ddPCR assay.

| Assay panel | Target pathogens and AMR genes |
| --- | --- |
| PilotBac-1 | *A. baumannii, E. coli, K. pneumonia, P. aeruginosa,* |
| PilotBac-2 | *E. faecalis, E. faecium, S. aureus, S. pneumoniae* |
| PilotBac-3 | *S. capitis, S. haemolyticus, S. hominis, S. epidermidis* |
| PilotBac-4 | *E. cloacae, P. mirabilis, S. marcescens, S. maltophilia* |
| PilotFungi-1 | *C. albicans, C. glabrata,* *C. parapsilosis, C. tropicalis* |
| PilotAMR-1 | *blaKPC, mecA, vanA, vanB* |

AMR, Antimicrobial Resistance

*blaKPC*, *Klebsiella pneumoniae* carbapenemase encoding gene;

**Supplementary Table 2**. Adjustment of antibiotic regimen based on ddPCR assay in the 10 non-survivors

| **Case** | **Age (years)**  **/sex** | **Comorbidities** | **Initial antibiotic regimen** | **Blood culture** | **Specific to pathogens^a^** | **First ddPCR assay**  **(Copy numbers)** | **Adjustment of the antibiotic regimen based on the first ddPCR assay** | **Surviving days** |
| --- | --- | --- | --- | --- | --- | --- | --- | --- |
| 2 | 86 / Male | COPD, Hypertension | Imipenem  + Daptomycin | *P. aeruginosa* | Yes | *P. aeruginosa* (40) | Yes  Piperacilin / tazobactam  + Colistin | 27 days |
| 3 | 56 / Male | Brain trauma | Piperacillin /tazobactam | *K. pneumonia* | No | *K. pneumonia* (35)  *P. aeruginosa* (36)  *C. parapsilosis* (83) | No | 5 days |
| 4 | 81 / Male | COPD | Imipenem  + Linezolid | *P. aeruginosa* | Yes | *P. aeruginosa* (152) | Yes  Meropenem  + Levofloxacin | 5 days |
| 6 | 87 / Male | Cerebral infarction | Cefoperazone /sulbactam  + Tigecycline  + Carpofungin | *K. pneumonia** | No | *K. pneumonia* (1300)  *A. baumannii* (2800)  *E. cloacae* (670)  *C. albicans* (30)  *blaKPC* (2600) | No | 6 days |
| 8 | 71 / Male | Prostate cancer, Hypertension,  Diabetes,  Immunosuppression | Meropenem  + Amikacin | *P. aeruginosa** | No | *P. aeruginosa* (2577)  *blaKPC* (2716) | No | 8 days |
| 9 | 75 / Male | Cervical spinal injury | Meropenem  + Tigecycline | *K. pneumonia* | Yes | *K. pneumonia* (2379)  *E. cloacae* (596) | No | 24 days |
| 13 | 64 / Male | Chronic kidney disease, Hypertension | Imipenem  + Moxifloxacin | *P. aeruginosa* | No | *P. aeruginosa* (2000)  *K. pneumonia* (50) | No | 5 days |
| 14 | 54 / Male | Bronchiectasis | Meropenem  + Colistin | *P. aeruginosa** | Yes | *P. aeruginosa* (3000) | No | 3 days |
| 15 | 88 / Male | Cerebral infarction | Ceftazidime /avibactam  + Colistin | *K. pneumonia** | Yes | *K. pneumonia* (230)  *P. aeruginosa* (28)  *blaKPC* (1000) | No | 6 days |
| 16 | 85 / Male | COPD, Hypertension | Imipenem | *K. pneumonia** | No | *K. pneumonia* (98)  *blaKPC* (3000) | No | 5 days |

COPD, Chronic obstructive pulmonary disease; *blaKPC*: *Klebsiella pneumoniae* carbapenemase encoding gene.

^a^ Assessment of empirical antibiotics specific to pathogens detected by blood culture.

* Multidrug-Resistant Organism, MDRO.

.
